# Planning nature in urbanized countries. An analysis of monetary and non-monetary impacts of conservation policy scenarios in the Netherlands

**DOI:** 10.1016/j.heliyon.2017.e00280

**Published:** 2017-03-30

**Authors:** Frans J. Sijtsma, Willem G.M. van der Bilt, Arjen van Hinsberg, Bart de Knegt, Martijn van der Heide, Hans Leneman, René Verburg

**Affiliations:** a Department of Economic Geography, Faculty of Spatial Sciences, University of Groningen, PO Box 800 9700 AV, Groningen, The Netherlands; b Department of Earth Science, University of Bergen, Allégaten 41, 5007, Bergen, Norway; c Department of Nature and Rural Areas, PBL Netherlands Environmental Assessment Agency, 2500 GH, The Hague, The Netherlands; d Wageningen University and Research, Alexanderveld 5, 2585 DB, The Hague, The Netherlands; e Deboerenstad, Tomatenstraat 201, 2564 CP, The Hague, The Netherlands; f Copernicus Institute of Sustainable Development, Utrecht University, Heidelberglaan 2, 3584CS, Utrecht, The Netherlands

**Keywords:** Economics, Environmental sciences

## Abstract

Planning and conserving nature areas are challenging tasks in urbanized and intensively used countries like the Netherlands. This paper supports decision making and public policy debate about these tasks in both an empirical and a methodological way. Empirically, we explore policy alternatives by determining the potential consequences of different nature policy scenarios in the Netherlands. Methodologically, we employ a mixed monetary and non-monetary evaluation method known as multi-criteria cost-benefit analysis (MCCBA). We evaluate four new future directions of Dutch nature policy that address four dominant stakeholder demands: biodiversity conservation, the provision of ecosystem services, recreational potential as well as economic gains. To balance compact presentation of evaluation outcomes on the one hand and information richness of results on the other, we distinguish between two impact indicator sets: three “headline” and ten “elaborate” indicators. Using these indicators we discuss the quantitative assessment of the four nature policy scenarios by comparing them to two other scenarios, reflecting the 2010 stand-still baseline situation (2010) as well as a reference policy (Trend). In total, we evaluate six scenarios; four present new directions and two reflect existing or recently (2010) halted practices. Our findings first of all show that even in an urbanized country like the Netherlands, with its intensive competition among land use functions, serious gains in national and international biodiversity are possible. Second, we find that it is doubtful whether stimulating the provision of regulating ecosystem services in a country which applies intensive and profitable agricultural techniques is beneficial. Other countries or areas that are less suitable for intensive agricultural practices may be more logical for this. Finally we demonstrate that increasing urban recreational green space − a common challenge for many urban areas − can only be achieved at relatively high costs, while it does not seem to lead to relatively high scores on nature appreciation. Nature appreciation seems to be served better by wilder nature than by park-like nature.

## 1 Introduction

Which natural areas should be protected, and the values on which protection should be based, are key questions for conservation management (Polasky et al., 2008; Borgström et al., 2013). To support decision making and wider policy debate, evaluation methods are used in which the different aspects of the policy choices are integrated and assessed (Gascoigne et al., 2011; Reichert et al., 2015). However, challenges arise in the use of these methods for conservation management as the impacts of different policy scenarios can be difficult to measure (Mea, 2005; Teeb, 2010; Nelson et al., 2009; Sijtsma et al., 2013b). From a measurement perspective, the merits of different nature policies and their accompanying ecosystem functions are typically described either by monetary (Teeb, 2010; Gascoigne et al., 2011) or non-monetary metrics (Mea, 2005; Ianni and Geneletti, 2010; Reichert et al., 2015), but rarely are they combined (Daily et al., 2009). Moreover, evaluation methods differ in the ways they reduce measured (sub-)scores to a limited set of indicators deemed to be useful to decision-makers, i.e. the process of aggregation. Most methods focus on aggregation to one summary indicator (Belton and Stewart, 2002; Ananda and Herath, 2009; Boardman et al., 2011; Zeleny, 2011), but aggregating to a small set of indicators, instead of only one, is rarely addressed (Janssen, 2001). Finally, the debate on the legitimisation of conservation policy is often also complicated by widely differing views and interests from various stakeholders (Seghezzo et al., 2011; Steinhäusser et al., 2015). Stakeholders tend to value the same interests and outcomes differently (Mustajoki et al., 2011; Teel and Manfredo, 2010; Adams et al., 2016). Therefore, through their methods and results, evaluations of nature conservation policies need to simultaneously incorporate a spectrum of views and respond to the substantive debate through measurement and indicators (Hermans et al., 2007).

The outlined questions and challenges to evaluation and planning of the protection of natural areas are very relevant and timely in the Netherlands, a strongly urbanized and intensively used country that has to comply with stringent national and European Union (EU) nature conservation legislation (European Commission (EC), 2011; Van Der Windt, 2012; Roodbol-Mekkes et al., 2012). Since 1990, Dutch nature policy has been dedicated to the formation of a National Ecological Network (NEN) of 728.500 hectares. A major policy challenge of NEN entails the planned conversion of approximately 275.000 hectares of productive agricultural land into new natural areas (Strijker et al., 2000; Jongman et al., 2004; de Jong et al., 2007).

The NEN’s primary focus to conserve biodiversity has led to conflicts of interest among stakeholders. As a consequence, 20 years after its conception, and over 75% of the project having been realised, the NEN was in jeopardy by 2010. Its ecological successes were put into question and the costs incurred and restrictions imposed on economic development were amplified. Certain stakeholders argued that greater emphasis ought to be placed on recreational opportunities, while others were, for instance, committed to strengthening the provision of ecosystem services − natural processes that are of key importance to human society (e.g. pollination). To further this debate, PBL Netherlands Environmental Assessment Agency designed four spatially-explicit scenarios for Dutch nature conservation in 2040. Each scenario addresses a dominant challenge to nature conservation − identified in stakeholder workshops (Van Oostenbrugge et al., 2010; Dammers and Evers, 2008).

This paper assesses the impacts of these four nature policy scenarios for the Netherlands, but also tries to address the broader methodological issue of evaluation of plans for nature protection. The evaluative workhorse which allows a broad stakeholder understanding is a mixed monetary and non-monetary evaluation method known as Multi-Criteria Cost-Benefit Analysis (MCCBA) (Sijtsma, 2006; Sijtsma et al., 2011; Sijtsma et al., 2013a; Sijtsma et al., 2013b). We present our evaluation in a compact ‘all together in one view’ fashion (Willcox, 1975), in which we succinctly outline the impacts and trade-offs between different scenarios for the benefit of decision-makers. This approach allows us to address the outlined methodological issue of balancing aggregation and information richness. We not only present results using mixed monetary and non-monetary indicators, we also vary the extent of aggregation. We present our findings using three ‘headline’ impact indicators (net costs and benefits, biodiversity and nature appreciation) as well as a more elaborate set of ten indicators (national and international biodiversity, nature appreciation, lack of urban recreational green space, nature management and investment costs, agricultural production, housing benefits, biomass energy, wood production and CO_2_ sequestration benefits).

Our main empirical objective is to calculate and interpret the impacts of different Dutch nature policy scenarios in a compact and understandable way. We will demonstrate that even in the densely populated Netherlands, with its intensive competition among land use functions, serious gains in national and international biodiversity are possible. Second, we will show that increasing the provision of regulating ecosystem services in a country which applies intensive and profitable agricultural techniques may be difficult. Finally we will find that increasing urban recreational green space − a common challenge for many urban areas − can only be achieved at a high cost, while other nature policy scenarios, involving wilder nature instead of park-like nature, score much higher on appreciation.

From a methodological stance, the implicit aim of this study is to show how MCCBA can give decision support by identifying trade-offs between nature policy scenarios. We argue that the insights gained from the presented Dutch case study may yield valuable empirical lessons for decision-support processes in similarly intensively used and/or urbanized areas.

## 2 Materials and methods

### 2.1 The evaluation method

**Fig. 1:**
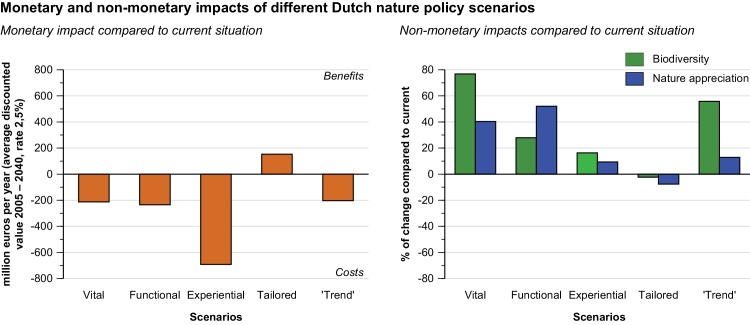
MCCBA results using 3 criteria for the monetary and non-monetary impacts of different scenarios for Dutch nature policy. The four scenarios are compared to the current (stand-still) baseline situation and the ‘*Trend*’ reference scenario.

Evaluations of nature related land use scenarios are commonly presented in either monetary (Gascoigne et al., 2011; Jenkins et al., 2010; Teeb, 2008) or non-monetary outcomes (Bouma 2002; Mea, 2005; Hermans et al., 2007; Wolfslehner and Seidl, 2010; Oikonomou et al., 2011; Mendoza and Martins, 2006). In this paper we mix both approaches, following e.g. Polasky et al. (2008), Nelson et al. (2009) and Sijtsma (2006). We do so by applying Multi-Criteria Cost-Benefit Analysis (MCCBA) (Sijtsma, 2006; Sijtsma et al., 2011; Sijtsma et al., 2013b), a mix of Cost Benefit Analysis (CBA) and Multi Criteria Analysis (MCA) (Fig. 1). This combined approach allows us to use non-monetary indicators, common in MCAs, whilst maintaining the analytical rigor of CBA monetization. The mixed approach places greater emphasis on quantification and less on valuation, downplaying the so-called “best” policy option (Viglizzo et al., 2012).

Evaluation methods need to strike a balance between aggregation and information-richness, i.e. between presenting results in a very compact, aggregated way and using indicators that convey easily comprehensible information for decision-makers and stakeholders. Aggregation to a compact view is a standard aim in any MCA, but achieving consensus on the weights of sub-indicators is often problematic (Belton and Stewart, 2002). Maximum aggregation, i.e. aggregation to one number, is also common in CBA but involves the monetization of *all* impacts (Boardman et al., 2011), which is likely to be contested (Clark et al., 2000), and compromises information-richness. The MCCBA used in this paper strives to be compact while conveying as much information as possible (Keeney and Raiffa, 1976; Janssen, 2001).

To help strike this balance, MCCBA strives towards standardized consensus-based indicators (Sijtsma, 2006). In this context, indicators should be comprehensible to most stakeholders and decision-makers, and all indicators should be seen as having a *minimal* relevance of being measured. In a first step, MCCBA uses the CBA technique to aggregate the easily monetized impacts to inform decision-makers about the costs and monetizable benefits of nature conservation. It is noteworthy, however, that MCCBA, as a rule, does not express biodiversity impacts in monetary units, given that no consensus has been reached on how to monetize this moral concern after decades of scientific discussion (Sijtsma et al., 2013b).

Other assessed impacts may be measured using either monetary or non-monetary indicators, the choice depending mostly on data-availability. The stance of MCCBA on this is comparable to Nelson et al., 2009; p. 5 who, when discussing their model, state that *“results can be reported in either biophysical or monetary terms, depending on the needs of decision-makers and the availability of data.”* In this paper, the balance of aggregation and information richness takes two forms. The first uses three headline indicators, the second uses a more elaborate set of 10 indicators.

**Fig. 2:**
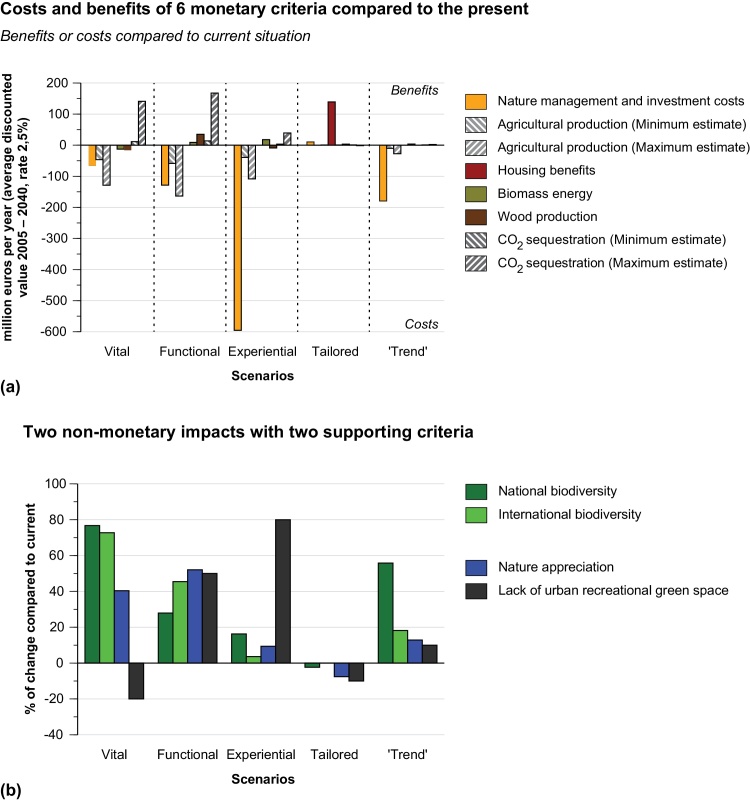
MCCBA results using 10 criteria. **a**: six monetary components (with two min-max estimates). **b**: Four non-monetary criteria. Note that a positive change in ‘Lack of urban recreational green space’ means there is more green space, i.e. there is a reduction in the shortage of green space.

Three was the minimum number of indicators we could arrive at in this evaluation (see also 2.3). Following long-established evaluation theory (Keeney and Raiffa, 1976; Boardman et al., 2011), there is obviously great merit in having as few indicators as possible, but we felt that a further aggregation of our three indicators to one final number would likely inhibit stakeholder problem understanding. These three mixed monetary and non-monetary indicators are all ‘understandable metrics’ (Mooney, 2010). The measurement scale of monetary costs and benefits, the scale of biodiversity changes and the scale of changes in the degree of appreciation of nature areas are metrics that ecologists, economists and different involved stakeholders can recognize and understand. We could also have chosen five indicators by adding international biodiversity alongside with national biodiversity or by adding the urban recreational shortages of green space alongside the appreciation of nature areas (see 2.3 below and Fig. 2b). However, we felt that this would cause redundancy or a partial double count (as to the two biodiversity indicators) and would introduce indicators which may not reflect end-points of well-being (as for the recreational shortages). The elaborate set of ten indicators first of all shows the (six) *main* components of the aggregate monetary indicator, components which highlight the *division* of costs and benefits to different stakeholders (e.g. the division between farmers and governments) (Fig. 2). On the non-monetary side, it shows two *competing* indicators for the two shown at the highest level of aggregation: these two extra indicators add important information, but at the price of introducing some redundancy.

### 2.2 The analysed normative scenarios

For the presented evaluation, we use four spatially-explicit land use scenarios for nature conservation in the Netherlands in 2040. These were designed by the Netherlands Environmental Assessment Agency (PBL) to guide the re-evaluation of Dutch nature policy in the aftermath of the 2008 financial crisis (Pbl, 2011; Pbl, 2013). Each of these scenarios addresses one dominant challenge for Dutch nature conservation, identified during stakeholder workshops (Van Oostenbrugge et al., 2010; Dammers and Evers, 2008). These challenges are: (**i**): halt the continuing loss of internationally important biodiversity, (**ii**) ensure an increase in and sustainable use of ecosystem services, (**iii**) enhance the recreational potential of nature near urban agglomerations, and (**iv**) allow the development of real estate in and nearby protected areas (Pbl, 2011; Pbl, 2013). The scenarios are summarized in Table 1 and elaborated in the text below.

We compare the four scenarios first to a baseline scenario that reflects the situation in the year 2010. This baseline entails a continued stand-still of Dutch nature policy as of 2010: no new nature protection areas are being realized in this scenario, no new directions are being taken. Second, the four new scenarios are not only compared to the outlined baseline, but also to a reference scenario called “Trend”. This second benchmark aims to further aid in the interpretation of the calculation of the costs and benefits of the future scenarios. This ‘Trend’ scenario envisions a continuation of the NEN policy that started in 1990 and was well underway in 2010.

This means that we evaluate six scenarios in total; four reflect new directions for Dutch nature policy and two reflect existing or recently halted practice around 2010. A detailed discussion of the underlying empirics of these scenarios would distract us from the main goal of this study. Thus, we only touch briefly on the empirical tools used to generate the discussed scenarios, instead referring to detailed background studies.

#### 2.2.1 Vital Nature: Halt the loss of internationally important biodiversity

By ratifying the EU Biodiversity Strategy (European Commission (EC), 2011), the Netherlands has committed itself to halting the continuing loss of biodiversity. Meeting this target is the main challenge of the *Vital Nature* scenario. Although the rate of biodiversity decline has slowed in recent years, the number of species on the various Red Lists has nevertheless increased between 1990 and 2004 (Cbs et al., 2013). The ongoing deterioration is mainly driven by pressures stemming from eutrophication, desiccation, climate change, fragmentation, and habitat loss (Reijnen et al., 2007).

In the *Vital Nature* scenario, conditions are optimised for the protection of species that are common in the Netherlands compared to other areas in the Atlantic biogeographic region (Van Hinsberg et al., 2011). To combat the impacts of the previously mentioned pressures, nitrogen deposition levels are to be reduced, groundwater tables raised, and areas of inter-connected natural areas created (Van Hinsberg et al., 2011; Pbl, 2011; Pouwels et al., 2016). In total, an additional 330.000 hectares of agricultural land are to be converted to new natural areas − compared to the used 2010 stand-still baseline (Table 1). In addition, 350.000 hectares of existing nature (small fragmented patches) will lose their protective status (Pbl, 2013). The scenario in total comprises 750.000 hectares (Table 1).

#### 2.2.2 Functional Nature: Ensure the sustainable use of natural resources

Since the publication of the Millennium Ecosystem Assessment (Mea, 2005) and The Economics of Ecosystems and Biodiversity synthesis (Teeb, 2010), significant research has focused on the merits of ecosystem services to express the value of natural capital (Mooney, 2010). Regulating (e.g. carbon sequestration and pollination) and provisioning services (e.g. biomass fuel and wood production), that are provided by nature and essential to human society (de Groot et al., 2010). Thus, implementing the concept of ecosystem services may provide an incentive for the sustainable use of natural resources. Several ecosystem services are currently in decline in the Netherlands due to increasingly mono-functional land use (Pbl, 2012).

*Functional Nature* aims to restore and strengthen the delivery of ecosystem services (Mea, 2005). In *Functional Nature*, the provision of selective ecosystem services is to be enhanced. These include carbon sequestration in peat lands, biomass harvest for energy production and wood production (Fig. 2a). Due to their ability to deliver ecosystem services, existing natural areas form the backbone of *Functional Nature*. Also, 84.000 hectares of purifying reed swamps are to be created to reduce surface water loads for Nitrogen and Phosphorus to legal limits (Sollie, 2007; Pbl, 2008b). A further 128.000 hectares of dried peat lands are to be turned into wetlands in order to halt CO_2_ emissions from degrading organic matter (Van der Bilt et al., 2012). These land claims make *Functional Nature* the largest scenario in terms of land use for nature, with a total surface area of 900.000 hectares (Table 1).

#### 2.2.3 Experiential Nature: enhance urban recreational nature

There is ample research to demonstrate that the Dutch public appreciates the presence of green areas in their environment, particularly for hiking and cycling. However, model calculations show that approximately 38% of Dutch households (Sijtsma et al., 2012b) experience a shortage of accessible natural areas in the vicinity of their residence. These shortages are expected to increase towards 2040, particularly around large cities (Mnp, 2006).

*Experiential Nature* aims to provide sufficient space for walking and cycling within 2.5–10 kilometres from the human living environment in 2040 (Van der Bilt et al., 2012). Future population and housing growth are estimated using the Global Economy growth scenario (Mnp, 2006). Calculations made with the AVANAR recreation model suggest that 119.000 hectares of new natural areas are required by 2040 to solve existing and projected shortages (de Vries et al., 2004; Pbl, 2012). Earlier studies have shown that diverse vegetation is appreciated by the Dutch public (de Vries et al., 2004); therefore the scenario evaluates areas comprising a mix of 1/3 forest, 1/3 water and 1/3 grasslands. In *Experiential Nature,* management of existing and new areas is tailored to recreational rather than ecological targets.

#### 2.2.4 Tailored Nature: real estate development in natural areas

To conserve biodiversity, the Netherlands is committed to stringent legislation at global (United Nations Convention on Biodiversity), European and national levels (Natura 2000), but these policies seem to restrict economic activity around Dutch nature. This is, for example, demonstrated by a decline in the number of farms close to Natura 2000 areas (Vonk et al., 2010), but nature areas near urban areas (‘green belts’) are also known to impose barriers against development of residential housing (e.g., Tang et al., 2007). In this scenario we focus on the latter − development of residential housing.

In the *Tailored Nature* scenario, friction between economy and ecology is tempered to enable economic residential development. Environmental and nature legislation will allow some residential development in and around existing natural areas. To assess the scale and type of land claims towards 2040, we use the Land Use Scanner model of Hilferink and Rietveld (1999), in combination with the Global Economy growth scenario (Mnp, 2006). In this scenario 30.000 hectares of existing natural area will be converted into residential areas and thus lost as a nature area (Table 1).

### 2.3 Evaluation indicators and calculations

As previously outlined, in the evaluation we attempt to balance information richness and aggregation by presenting our findings using sets of 3 and 10 impact indicators, respectively. As with any evaluation, we strive to ensure that our evaluative indicators account for the (social) values at stake, which, for instance, have been identified by stakeholders and incorporated into the assessed scenarios. It is thus critical to obtain measurable information which addresses these values and concerns (Keeney, 1996). The details of the selected indicators and background information on performed calculations (e.g., applied models) are outlined below.

### 2.4 Costs

Costs are a common concern to any evaluation, but given the budget cuts in the aftermath of the 2008 financial crisis, managing the financial costs of nature conservation has become a high priority. We therefore argue that investment and maintenance costs are salient indicators. To calculate these costs, we employ the large database compiled by de Koeijer et al., 2006; de Koeijer et al., 2008 and Leneman et al. (2010). In this database, relevant costs are matched with types of nature and regional differentiation of biophysical effects (e.g., nitrogen deposition and desiccation). As is common to CBAs, costs are aggregated to a Net Present Value (NPV). A time period of 30 years (2010–2040) is used in conjunction with a 2,5% discount rate, in accordance with the official Dutch government prescription (Kamerbrief, 2007). The total NPV is dependent on the length of the assessed period, but this may make the NPV less easy to interpret by non-economists. With the sting of austerity still present, which mostly relate to yearly outlays, we express the total NPV as an average value per year.

### 2.5 Agricultural production value

As mentioned in Section 2.2.4, agriculture is the main land use type affected by nature conservation policies in the Netherlands. We therefore argue that impacts on agricultural production values should also be quantified as part of the social monetary indicators of the MCCBA. In Table 2 we show that the main value at stake is the production value of agricultural land, reflected by the indicator (loss of) agricultural production. In conjunction with national productivity figures (Leneman et al., 2013), we have used GIS maps with standardised agricultural production size information to calculate different agricultural productivity between areas We performed an assessment of the lost net added value using data from the Dutch Farm Structure Survey (Landbouwtelling) which specifies type and size (in economic output terms) of farms and location (Cbs, 2009). The analysis assigns agricultural production value (NPV) to the three primary factors of production: land, labour and capital. The value associated with land is assumed to be permanently lost. Minimum and maximum estimates were calculated for changes in labour and capital.

Following the procedure described in Strijker et al. (2000), the lowest estimate of production loss is represented by the gradual shift of labour and capital to other economic sectors over the course of 10 years. For the maximum estimate of production loss, we assume that 50% of agricultural labour and capital cannot be effectively employed in other economic sectors (de Blaeij and Reinhard, 2008; Geerling-Eiff and Van Der Meulen, 2008). In addition, we also take the indirect costs of agribusiness activity (e.g., suppliers, etc.) into account, roughly doubling the primary agricultural production loss estimates. The resulting impacts are calculated as an NPV over a 30-year period (2010–2040), with a yearly discount rate of 2,5%. This number is then expressed as an average value per year (as with costs) (see Table 2).

### 2.6 Selected ecosystem services

With regard to ecosystem services, we concentrate on services that **I**) can be provided without human intervention (management) and **II**) are known to be able to contribute to policy targets in the Dutch context (Tolkamp et al., 2007; Pbl, 2008a; Nabuurs and Schelhaas, 2002). This selection includes carbon sequestration, biomass energy generation and wood production (Leneman et al., 2013). In our analysis, we only calculate carbon sequestration for forested areas, as amounts sequestered by grass and heathlands are negligible (Van der Bilt et al., 2012). Since wood density is strongly related to growth rate and hence carbon storage, we classify tree species into five wood density groups using the CO_2_ fix model (Nabuurs and Schelhaas, 2002). Moreover, we specify a certain percentage of coverage of the five types for each nature target type over the Nature Outlook 30-year time span for our analysis (Van der Bilt et al., 2012). For monetary valuation of carbon sequestration, data are compared between the tradable emissions system (Zhang and Wei, 2010) and that of forest certificates (Schelhaas et al., 2002). Resulting valuation data indicates a wide price range, from €5–50 per/ton carbon (C). To calculate the potential of biomass energy, we use the same CO_2_–fixation-model approach to assess both biomass and soil carbon increments in new forests. Only trunk wood is used for wood supply, whereas branch wood can be used as biomass energy. Mowing grasslands and managing heath lands also provides additional biomass. With regard to the monetary valuation of biomass energy, we use market prices in 2010 of pit coal and natural gas and recalculate them in relation to their energy content; prices vary from €1,70 per/GJ to €5,00 per/GJ. We then use these figures to value the *energy* content of biomass (minimum and maximum values). We assume that wood production is supplied exclusively from existing forests that were characterized as mature (full-grown) in baseline year 2010 (Schelhaas et al., 2002 and references therein).Only trunk wood is used for wood supply. The monetary value of wood is derived from market data (in 2010 at around €60 per/m^3^–see Van der Bilt et al. (2012)).

### 2.7 Housing benefits

We only calculate housing benefits for the *Vital* and *Tailored* nature scenarios (section 2.2). While the latter scenario encourages real estate development in and around natural areas, the former *in principle* allows for housing to be built in protected and attractive natural areas of limited international significance (2.2.1). We focus on assessing the most prominent differentiating effect: the possible capital gains related to real estate development opportunities arising from a loosened protection regime of natural areas in the *Tailored Nature* scenario (section 2.2.4). As described in 2.2.4, we apply the Land Use Scanner model to assess the scale and location of future real estate development using two economic scenarios, projecting high and low growth rates (Hilferink and Rietveld, 1999; Mnp, 2006). We identify three likely classes of built-up area in formerly protected natural areas: rural, green-urban and urban. For these classes we use densities of 6, 7 and 22 houses per hectare, respectively, after MNP (2006). Non-built up area is assumed to retain its natural state. The projected potential price gains range from 3–6% for the urban area (Bolitzer and Netusil, 2000; Poudyal et al., 2009), 4–8% for green-urban areas (Luttik, 2000), and 9–12% for rural areas (Visser et al., 2006; Nicholls and Crompton, 2005). We use the range showing the highest estimate of these price gains. Research using the Hotspotsmonitor (Langers et al., 2013; Sijtsma et al., 2013a), a survey tool that measures landscape appreciation, suggests that housing value increments are much higher in *appreciated natural areas* than in green or nature areas which are merely classified as such in a land-use sense (Daams, 2016; Daams et al., 2016). Since the projected new housing in the assessed scenarios is likely to be built in such highly appreciated natural areas, our use of the highest price gain estimates seems to be justified. We convert our capitalised values to an annual housing rent at a discount rate of 2.5%, using a 30-year depreciation period.

### 2.8 Biodiversity impacts

Due to the ratification of the EU Biodiversity Strategy to 2020 (European Commission (EC), 2011), we foresee that biodiversity conservation will remain a focal point of Dutch nature policy in the coming decades. In accordance with set policy targets, we focus on both the status of species and the quality of ecosystems (EEA, 2007). To assess the effects of the different scenarios on biodiversity, we use the Model for Nature Policy (MNP) (Pouwels et al., 2016). The MNP successively models habitat suitability and persistence for sets of protected species. In the present paper we use the number of sustainably protected species as an indicator (Table 2), but the model is also closely related to ecosystem intactness (Pouwels et al., 2011; Pouwels et al., 2016). As previously mentioned in 2.2.1, the *Vital Nature* scenario focuses on habitats and species that are relatively common in the Netherlands compared to other areas of the Atlantic bio-geographical region (de Knegt et al., 2011a); this indicator is part of our 10 indicator set. However, to improve stakeholder relevance, we use a more general biodiversity indicator for the headline set. Policy targets have also recently tended to focus on the protection of national biodiversity (Turnhout et al., 2007). Therefore, we use a headline indicator that measures the conservation status for species which are important from a national viewpoint (Bal et al., 2001) (Table 2, *national biodiversity*). Specifically, this indicator assesses a set of 331 species of terrestrial vascular plants, breeding birds and butterflies which respectively comprises 41%, 79% and 92% of the Dutch target species of these taxonomic groups (de Knegt et al., 2011a).

### 2.9 Recreational value

Here, we do not value the appreciation of nature (e.g., aesthetic or recreational) in monetary terms, because we have not found suitable monetary data or model(s) with which to parameterize this indicator for the assessed scenarios. Instead, we use two non-monetary indicators. The first indicator is drawn from the previously mentioned Hotspotmonitor (de Vries et al., 2013; Sijtsma et al., 2012a; Sijtsma et al., 2013a), an interactive map-based online survey tool that measures the attractiveness of natural places. We use the so-called *Hotspot index* from this tool, which sums the number of times areas have been selected as highly attractive by respondents, compared to the chance of randomly selecting an area (Sijtsma et al., 2013a). The resulting appreciation index (Table 2), part of the three headline indicator set, is multiplied by the different sets of hectares in different scenarios to give an appreciation score for each scenario (Sijtsma et al., 2013a).

Our second non-monetary indicator is used to evaluate the recreational appreciation of natural areas and complements the appreciation index. This indicator is built on output derived from the AVANAR recreation model (de Vries et al., 2004), estimating the shortage of recreational green areas. The AVANAR model evaluates the surface of green area available for recreational walking and biking (the most popular recreational activities in the Netherlands (Nbtc-Nipo, 2013)) in our study, relative to the desired amount, which is determined by spatial distribution of population and population density (Sijtsma et al., 2012b). Table 2 provides an overview of all the indicators used in the scenarios and the main sources of the calculations.

## 3 Results and discussion

### 3.1 The use of three indicators

As explained above, we present results using two sets of indicators, one with 3 and one with 10 indicators. Since the main aim of the presentation of the results is the comparison between the different scenarios, we present these results in graphs and not in tables (Vessey, 1991). Fig. 1 shows the aggregation to three headline indicators, comprising the monetary valued net costs and benefits outcome, the impact on biodiversity and the impact on nature appreciation. Fig. 1 displays conservative estimates, and includes *maximum* estimates of agricultural *costs* and *minimum* estimates of ecosystem *benefits*. The non-monetized effects are represented by an index, where the 2010 stand-still baseline situation serves as a reference point (0), thereby highlighting the differences between the present and the assessed future scenarios.

The left-hand side of Fig. 1 shows that the *Vital* and *Functional* nature scenarios have net costs comparable to the benchmark *Trend* scenario, with net costs of around €200 million per year. The costs of three of these five scenarios are indeed comparable, although they differ notably in spatial configuration and ambition. *Experiential* nature on the other hand, is far more expensive, and costs three times as much as the afore-mentioned scenarios, with annual net-costs exceeding €600 million. This shows that finding the solution to existing and forecasted recreational shortages is very costly. Finally, as for the monetarily valued costs and benefits, Fig. 1 highlights that *Tailored* nature, by relaxing nature protection and selling natural land for residential real estate development, is the only scenario that is predicted to generate net monetary benefits.

The right-hand side of Fig. 1 reveals that the scenario *Vital* nature succeeds in optimizing the conservation status of biodiversity and outperforms the *Trend* benchmark, with a projected 76% improvement compared to the present. The *Functional* nature scenario also delivers significant biodiversity benefits, suggesting strong synergies between biodiversity conservation and ecosystem services. Biodiversity gains in *Functional* nature benefit in particular from increases in natural coastal nature and water-rich areas that sequester carbon and retain water. The evaluation result thus uncovers the presence of synergistic benefits between biodiversity and climate policies.

Notwithstanding the relaxation of protection regimes, differences in biodiversity scores between *Tailored* nature and the present stand-still situation are minimal. It should be noted, however, that the applied biodiversity indicator can possibly mask negative population trends which may render species populations unsustainable over longer timescales (de Knegt et al., 2011a; de Knegt et al., 2011b). Fig. 1 also shows that the realisation of *Experiental* nature would lead to modest biodiversity gains compared to most of the other scenarios. The implication here is that synergies between recreational potential and biodiversity are not very strong. Furthermore, our results for *Experiental* nature also clearly demonstrate that recreational potential and appreciation are not per se coupled, as both *Vital* and *Functional* nature score substantially higher on this criterion. The higher scores by the latter two are driven by the restoration of highly appreciated ecosystems such as dunes and heathlands (results of separate ecosystems are not shown, but see de Vries et al. (2013) for scores of different nature types). In contrast, the new park-like nature envisioned for *Experiential* nature is mostly optimised to cater to a maximum number of cyclists and walkers per hectare. Results show that in terms of appreciation, this type of urban park-like nature cannot compete with the ‘wild’ ecosystems found in the *Vital* and *Functional* nature scenarios (Bijker and Sijtsma, 2017).

Not surprisingly, *Tailored* nature is the only scenario that is less appreciated than the continuation of the present stand-still situation, due to the encroachment of residential areas into natural spaces. The privately-owned plots used for housing impose limits on accessibility to nature for the general public and therefore lead to a loss of nature appreciation (on the right of Fig. 1). New benefits are enjoyed too, but only exclusively by the few people that are able to purchase land, as reflected in the monetary benefits (shown on the left side of Fig. 1).

### 3.2 Elaborated results and discussion using 10 indicators

The aggregated headline indicators discussed above provide us with an overview, but at certain points they may lack the information richness required to design effective policy strategies. To provide decision-makers and other stakeholders with more information on the trade-offs and synergies between the scenarios, we now turn to our evaluation results using a wider set of 10 indicators, as shown in Fig. 2a,b. As discussed the choice of 10 is not random: this set represents the biggest separate components for the monetary impacts, while for every non-monetary impact one alternative key indicator was used. Fig. 2a depicts monetary impacts in six separate categories: **1**) management and investment costs, **2**) losses and gains in agricultural production, **3**) housing benefits, and benefits accrued from **4**) biomass energy, **5**) wood production, and **6**) CO_2_ sequestration. It should be noted, however, that both **2**) and **6**) are rather uncertain, and for this reason we include maximum and minimum estimates. Fig. 2b gives results for four non-monetary indicators, illustrating first of all not only the changes in national biodiversity, but also in international biodiversity, and second not only nature appreciation but also lack of urban recreational green space.

These more disaggregated results shed more light on the performance of the scenarios. The detailed results in Fig. 2a,b exemplify that even in the densely populated Netherlands, with its intensive competition among land use functions, it is still possible to achieve high scores on different nature policy targets. For example, high scores for international biodiversity (*Vital*) or high scores for the improvement in urban green space (*Experiential*). However, these results also show that the search for solutions to all of the identified challenges, reaching their potential simultaneously, is not easy. Next, we discuss these trade-offs and synergies; first, the examination of the distribution of monetary costs and benefits; second, the synergistic benefits between national and international biodiversity; and third, the trade-offs among ecosystem services.

### 3.3 Distribution of monetary cost and benefits

Fig. 2a demonstrates the major differences in costs for nature management and investment. These costs rest mainly with government(s) either directly or through subsidies. For the *Trend* scenario these costs approach €200 million per year and represent the bulk of the costs. The *Functional* scenario has far lower costs (around €120 million) while *Vital* costs much less, around €50 million per year, reflecting the fact that these two scenarios involve larger self-managing and wilder natural systems than the *Trend* scenario. However, although the structural costs of the *Vital* and *Functional* nature scenarios for nature management are lower for government(s) and may be considered to be appealing for that reason, Fig. 2a shows that for society at large, a bigger price is paid by farmers who lose the added value of their crops due to large-scale conversion of agricultural land into new natural areas. The net sum of these two cost elements is easily comparable for the three scenarios, as shown in Fig. 1: around €200 million per year. But thanks to the more detailed exposition in Fig. 2a, overall results contain strikingly different costs for the different stakeholders.

Fig. 2a also shows that costs in the *Experiential* scenario are mainly nature management and investment: costs generally paid by governments. The relatively high costs were already clear, but Fig. 2 now highlights the origin of these costs. The fact that these large costs rest mainly with one type of stakeholder, i.e. government(s), and that governments face myriad challenges concerning budget cuts, strongly lowers the feasibility of this scenario. Fig. 2a also shows that the benefits of the *Tailored* nature scenario involve no other substantial costs and mainly involves housing benefits.

As for housing benefits, in principle, the *Vital* nature scenario also includes housing benefits: with the anticipated focus on international biodiversity areas, those areas which merely served *national* biodiversity targets, can possibly be used for other functions, such as allowing housing in these nature areas. These potential benefits − comparably calculated as carried out for the *Tailored* scenario − would be approximately €175 million. However, these benefits are not included in the results, because we think this is not consistent with the scenario. While in the *Tailored* nature scenario it is logical and consistent to relinquish collective nature for private housing, in the *Vital* scenario there is a (very) strong commitment to nature and biodiversity, albeit broadly focusing on international biodiversity. Thinking within such a scenario, and given the accompanying public spirit, it seems neither logical nor consistent to *simply* hand over public nature areas that are no longer needed for international biodiversity to private housing interests; and this is precisely what the €175 million suggests. However, this still could happen *to some unknown extent*. It could happen after careful deliberation or it could perhaps happen only in a few places. These housing benefits therefore offer some extra incentive to realize the *Vital* nature scenario.

### 3.4 Biodiversity synergies

In Fig. 2b, the different scenarios demonstrate a strong difference in performance that was not visible using the three headline indicators: the impact of different scenarios for national versus international biodiversity was not clear at that point. Results show that *Vital* nature has considerable synergistic benefits between both types of biodiversity, but this is not the case for all scenarios. *Functional* nature also scores well on international biodiversity, but does not have a comparable performance on national biodiversity. The *Trend* scenario performs the other way around: it is weak on international biodiversity and strong on national biodiversity. This spectrum of scores shows that even in the highly urbanised and intensively-used Netherlands, serious gains in biodiversity are possible. Furthermore, Fig. 2b demonstrates once again that, even within a fairly restricted domain of performance, in this case biodiversity performance, there may be synergistic benefits *or* conflicts between two scores for a single scenario.

### 3.5 Trade-offs between ecosystem services

The monetary benefits of *Functional* nature may be seen as modest, considering that several ecosystem services have been monetized. With regard to the benefits of selected ecosystem services, the disaggregated results of Fig. 2a shows a range of uncertainty about the monetary benefits of ecosystem services: for agricultural production losses and CO_2_ sequestration, minimum and maximum estimates are shown. The estimates of the benefits of carbon sequestration are highly contingent on external developments such as global energy prices and the implementation of carbon trading schemes. The estimates of agricultural production loss depend on the extent to which factors of production (labour and capital) can be employed elsewhere in the economy. The comparatively low costs of the large *Functional* and *Vital* nature scenarios (shown in Fig. 1) may now be explained by the (minimum estimates) benefits from CO_2_ sequestration that offset the loss of agricultural production capacity in new natural areas. Many combinations of minimum and maximum estimates are feasible. However, if we combine minimum costs to minimum benefits or maximum costs to maximum benefits, the benefits of regulating ecosystem services cannot compensate for the loss of provisioning services like food production using the Dutch intensive agricultural practices. This result suggests the need for spatial specialization of areas in the provisioning of ecosystem services that go beyond the national scale of the Netherlands. In other words, these results suggest that more potential may accrue by focusing on ecosystem services in European areas that are not used or unsuitable for intensive agricultural practices.

Fig. 2b shows two indicators for cultural ecosystem services: the non-monetary indicator for the appreciation of nature and the indicator for the lack of recreational green space around urban areas. Results from Fig. 2b suggest that conflicts may arise, even within this restricted cultural services domain: a scenario that scores well on the one may not on the other. The appreciation indicator is the broader of the two indicators and directly measures positive appreciation for specific nature at the national scale; it may point to areas highly appreciated near urban areas and also to areas valued for holiday purposes or for incidental visits (Bijker and Sijtsma, 2017). The other indicator measures the available area for nature in the daily urban system (the area around a city in which daily commuting takes place): this indicator is pertinent for urban areas with little green per capita, and concerns a fairly frequent use of nature. Fig. 2b demonstrates that *Experiential* nature largely solves the problem of the lack of urban recreational green space in the Netherlands. This common challenge for many urban areas (Tzoulas et al., 2007; Sijtsma et al., 2012b) can thus be faced squarely; although − as we have seen − at a high cost. However, the type of nature that is created is not the most appreciated at the national scale: the indicator measuring appreciation of nature only slightly increases (de Vries et al., 2013). Scenarios not focused on nearby urban green space score much higher on appreciation.

## 4 Conclusions

MCCBA, like many Multi-Criteria Analysis approaches (Belton and Stewart, 2002; Ananda and Herath, 2009) is not designed to give the “best” choice option, but instead emphasizes the enhancement of problem understanding. The MCCBA evaluation carried out for this analysis has identified the scale of major impacts and important trade-offs for different nature policy scenarios in the Netherlands. Our study contributes to the policy debate by highlightingthat a range of choices and possible alternatives become apparent when applying monetary and non-monetary impact indicators whilst assessing nature (Zeleny, 2011). What lessons have we learned from the alternative scenarios?

### 4.1 Lessons learned from the scenarios

Two of the five scenarios seem to have little attractiveness. The *Experiential* scenario is extremely costly and performs only well with respect to the challenge for which it was designed: increasing urban green space. But neither for biodiversity nor appreciation does it have much to offer. *Tailored* nature brings economic benefits to a happy few but is also quite narrow in its results: it gives higher house prices in proximity to nature, but conveys no serious performance on any other aspect, except government budget(s).

The remaining options to consider as the most serious policy alternatives are *Functional*, *Vital* and *Trend*. These three perform more broadly than *Experiential* and *Tailored*, and at a reasonable cost. The *Functional* nature scores in the monetary part strongly depend on the estimates for the value of CO_2_ sequestration. If we do not take the highest CO_2_ sequestration values of the range, then stimulating the provision of regulating ecosystem services in a highly urban country like the Netherlands, which also applies intensive and profitable agricultural techniques, does not seem sensible. Results suggest that other less intensively used areas outside the Netherlands are likely better candidates for this type of scenario if the goal is to enhance regulating ecosystem services. However, the *Functional* nature scenario performs well on international biodiversity, on appreciation and on increasing urban green space. The *Vital* nature scenario also performs strongly on both national and international biodiversity as well as appreciation. Given these conclusions, it may now be easier to understand the societal and political discontent with the *Trend* scenario, which led to the stand-still and lack of direction in Dutch nature policy around 2010. Continuation of the *Trend* scenario leads to robust performance on national biodiversity, but poor scores with regard to international biodiversity, nature appreciation and reducing lack of urban green space. Our results suggest that the *Trend* scenario is not attractive, given benefits outlined for the other scenarios.

Clearly, new directions for Dutch nature policy need not be restricted to a choice among the scenarios sketched here. Other scenarios can be constructed and thought-through, combining the best of different worlds instead of addressing specific challenges. A new policy alternative emerging from the presented evaluation results is a combination of the *Vital* and *Tailored* nature scenarios. Such a new alternative would open the door to the combination of short and long-term interests by allowing, for example, a housing development to take place in areas that are not of fundamental importance to the conservation of internationally-important nature. The economic Willingness To Pay (e.g. Wertenbroch and Skiera, 2002) for living in these areas may be used to co-finance nature conservation in other areas. A combination of *Vital* with *Functional* is also worthy of consideration; both scenarios share a strong performance on appreciation and national biodiversity. *Functional* is relatively weak on international biodiversity, whereas *Vital* scores strongly. And *Vital* scores weak on increasing urban green space, where *Functional* is strong. Further analysis of the possibilities of integration might lead to the development of a policy with the broadest spectrum of performance.

### 4.2 The methodological lessons

From the methodological standpoint, we may conclude that the main results of this study can be presented in a compact format using only three indicators measured on cardinal scales. Contrary to the often used monetization of non-use values commonly applied in CBA, which allows a one-indicator presentation of results, these three mixed monetary and non-monetary indicators are all ‘understandable metrics’ (Mooney, 2010). The measurement scale of monetary costs and benefits, the scale of biodiversity changes and the scale of changes in the degree of appreciation of nature areas are metrics that ecologists, economists and different involved stakeholders can recognize and understand. Still, while a compact aggregation has great merit in evaluation, one might argue that the breadth of the results shown through the use of 10 indicators represents an indispensable extra source for solid problem-understanding and well-founded consideration of nature policy trade-offs. Therefore, we conclude that in the highly multi-disciplinary process of land-use evaluation, explicit methodological flexibility in balancing aggregation and information richness certainly seems to be a valuable asset.

## Declarations

### Author contribution statement

Frans Sijtsma, Willem Van der Bilt, Arjen Van Hinsberg, Bart De Knegt, Martijn Van der Heide, Hans Leneman, René Verburg: Analyzed and interpreted the data; Contributed reagents, materials, analysis tools or data; Wrote the paper.

### Funding statement

This research did not receive any specific grant from funding agencies in the public, commercial, or not-for-profit sectors.

### Competing interest statement

The authors declare no conflict of interest.

### Additional information

No additional information is available for this paper.

## Figures and Tables

**Table 1:** Description of the 4 tested normative scenarios and reference ‘*Trend*’ scenario.

	Normative scenarios				Benchmark scenario
Name	*Vital Nature (i)*	*Functional* *Nature (ii)*	*Experiential Nature (iii)*	*Tailored Nature (iv)*	*Trend*
Objective	Halt international biodiversity loss	Use of regulating ecosystem functions	Enhance nature’s recreational potential	Capacity for economic development	Mix
Measures	-Protection of areas with high international biodiversity	-Protection of natural areas which deliver selected ecosystem services	-Protection of appreciated and used areas	-Residential use of natural areas	-Protection of current areas
	-Additional areas to reduce fragmentation	-Additional areas to improve ecosystem services	-Additional green areas around cities	-No extension of natural areas	-Realisation of the National Ecological Network (NEN)
	-Optimal environmental conditions	-Conditions needed for ecosystem functioning	-Conditions needed for conservation	-No improved conditions	-Optimal environmental conditions
	-Management for restoring natural processes	-Management for delivering functions	-Management for recreational use	-Management in remaining natural areas	-Management for nature conservation, recreation and use
Additional area^1^	+330.000 ha	+320.000 ha	+120.000 ha	-30.000 ha^3^	+150.000 ha
Total area	750.000 ha^2^	900.000 ha	700.000 ha	550.000 ha	730.000 ha

1 Areas in hectares.

2 Not all current natural areas are part of *Vital Nature*; some new nature is realised on new land.

3 Additional area is transformed from natural areas to gardens.

**Table 2:** Impact indicator overview, indicating headline indicators with a (*) and highlighting each of the “elaborate” ten indicators in italics.

Indicators		Main sources for the calculations
Monetary indicators (*)		
*Management and investment costs*	Net Present Value (NPV) of monetary costs and benefits over 2010–2040 (discount rate 2,5%), expressed as an average yearly amount.	Leneman et al., 2013; de Koeijer et al., 2006; de Koeijer et al., 2008; Leneman et al., 2010.
*Agricultural production*	Net Present Value (NPV) of agricultural production loss (or gain), including losses (or gains) in agribusiness; losses over 2010–2040 (discount rate 2,5%), expressed as an average yearly amount.	Maximum estimate: Leneman et al., 2010; Leneman et al., 2013. Minimum estimate: Leneman et al., 2013, plus replacement procedure from Strijker et al., 2000.
*Ecosystem services:*		
* Wood production*	Euro NPV (average per year) of wood production.	Tolkamp et al., 2007; Pbl, 2008a; Nabuurs and Schelhaas, 2002; Leneman et al., 2013.
* Biomass energy*	Euro NPV (average per year) of Biomass Energy from new forests, grass and heath lands.	
* CO_2_ sequestration*	Euro NPV (average per year) of increased CO_2_ capture of new forests (minimum and maximum estimate).	
*Housing benefits*	Euro NPV (average per year) of housing benefits due to living in nature areas.	Daams, 2016; Luttik, 2000; Hilferink and Rietveld, 1999. Bolitzer and Netusil, 2000; Poudyal et al., 2009; Visser et al., 2006; Nicholls and Crompton, 2005.
**Non-monetary impacts**		
National biodiversity(*)	% of national target species sustainably protected (difference 2010–2040)	Pouwels et al., 2011; Pouwels, 2000; Verboom et al., 2001; Pouwels et al., 2016.
*International biodiversity*	% of international target species sustainably protected (difference 2010–2040)	
Nature appreciation(*)	% gain in appreciated natural areas (difference 2010–2040)	de Vries et al., 2013; Sijtsma et al., 2013a; Daams, 2016; Daams et al., 2016.
*Lack of or urban recreational green space*	% decrease in the lack of urban green space (difference 2010–2040)	de Vries et al., 2004; Sijtsma et al., 2012b.

## References

[bib0005] Adams V.M., Pressey R.L., Álvarez-Romero J.G. (2016). Using Optimal Land-Use Scenarios to Assess Trade-Offs between Conservation, Development, and Social Values. PloS one.

[bib0010] Ananda J., Herath G. (2009). A critical review of multi-criteria decision making methods with special reference to forest management and planning. Ecol. Econ..

[bib0015] Bal D., Beije H.M., Fellinger M., Haveman R., Van Opstal A.J.F.M., Van Zadelhoff F.J. (2001). Handboek natuurdoeltypen 2e geheel herziene druk.

[bib0020] Belton V., Stewart T.J. (2002). Multiple criteria decision analysis: an integrated approach.

[bib0025] Bijker R.A., Sijtsma F.J. (2017). A portfolio of natural places: Using a participatory GIS tool to compare the appreciation and use of green spaces inside and outside urban areas by urban residents. Landsc. Urban Plan..

[bib0030] Boardman A.E., Greenberg D., Vining A., Weimer D. (2011). Cost-benefit analysis: Concepts and practice.

[bib0035] Bolitzer B., Netusil N.R. (2000). The impact of open spaces on property values in Portland: Oregon. J. Environ. Manage..

[bib0040] Borgström S., Lindborg R., Elmqvist T. (2013). Nature conservation for what? Analyses of urban and rural nature reserves in southern Sweden 1909–2006. Landsc. Urban Plan..

[bib0045] Bouma J. (2002). Land quality indicators of sustainable land management across scales. Agric. Ecosyst. Environ..

[bib0050] Cbs (2009). Landbouwtelling.

[bib0055] Cbs, Pbl, Wur (2013). Compendium voor de Leefomgeving.

[bib0060] Clark J., Burgess J., Harrison C.M. (2000). I struggled with this money business: Respondents’ perspectives on contingent valuation. Ecol. Econ..

[bib0065] Daams M.N. (2016). Rethinking the economic valuation of natural land. Analyses of how deeply people value nature in rural areas and in cities, PhD Thesis.

[bib0070] Daams M.N., Sijtsma F.J., Van Der Vlist A.J. (2016). The effect of natural space on nearby property prices: accounting for perceived attractiveness. Land Econ..

[bib0075] Daily G.C., Polasky S., Goldstein J., Kareiva P.M., Mooney H.A., Pejchar L., Shallenberger R. (2009). Ecosystem services in decision making: time to deliver. Front. Ecol. Environ..

[bib0080] Dammers E., Evers D. (2008). Beyond heuristics: Applying scenarios to European territorial development. Tijdschrift voor economische en sociale geografie.

[bib0085] de Blaeij A., Reinhard S. (2008). Een waterpark als alternatief; MKBA aanleg multifunctioneel helofytenfilter op Waterpark Het Lankheet.

[bib0090] de Groot R.S., Alkemade R., Braat L., Hein L., Willemen L. (2010). Challenges in integrating the concept of ecosystem services and values in landscape planning, management and decision making. Ecol. Complex..

[bib0095] de Jong J.J., Van Dobben H.F., Wamelink G.W.W., Van Wijk M.N. (2007). Decreasing deposition will reduce costs for nature management. J. Nat. Conserv..

[bib0100] de Knegt B., Van Hinsberg A., Van Der Bilt W.G.M., Van Eupen M., Pouwels R., Reijnen M.S.J.M. (2011). Ecologische effectberekening Natuurverkenning.

[bib0105] de Knegt B., Van Eupen M., Van Hinsberg A., Pouwels R., Reijnen M.J.S.M., de Vries S., Van Der Bilt W.G.M., Van Tol S. (2011). Ecologische en recreatieve beoordeling van toekomstscenario’s voor natuur op het land.

[bib0110] de Koeijer T.J., Van Bommel K.H.M., Van Esbroek M.L.P., Groeneveld R.A., Van Hinsberg A., Reijnen M.J.S.M., Van Wijk M.N. (2006). Methodiekontwikkeling kosteneffectiviteit van het natuurbeleid; de realisatie van het natuurdoel Natte heide Wageningen.

[bib0115] de Koeijer T.J., Van Bommel K.H.M., Clement J., Groeneveld R.A., de Jong J.J., Oltmer K., Reijnen M.J.S.M., Van Wijk M.N. (2008). Kosteneffectiviteit terrestrische Ecologische Hoofdstructuur; Een eerste verkenning van mogelijke toepassingen Wageningen.

[bib0120] de Vries S., Buijs A.E., Langers F., Farjon H., Van Hinsberg A., Sijtsma F.J. (December 2013). Measuring the attractiveness of Dutch landscapes: Identifying national hotspots using Google Maps. Appl. Geogr..

[bib0125] de Vries S., Hoogerwerf M., Regt W.J. (2004). AVANAR: een ruimtelijk model voor het berekenen van vraag-aanbodverhoudingen voor recreatieve activiteiten; basisdocumentatie en gevoeligheidsanalyses. (AVANAR: a spatial model for the calculation of demand-supply ratios for recreation activities; basic documentation and sensitivity analysis) [in Dutch].

[bib0130] EEA (2007). Halting the loss of biodiversity by : Proposal for a first set of indicators to monitor progress in Europe.

[bib0135] European Commission (EC) (2011). Our life insurance, our natural capital: An EU biodiversity strategy to 2020 Brussels.

[bib0140] Gascoigne W.R., Hoag D., Koontz L., Tangen B.A., Shaffer T.L., Gleason R.A. (2011). Valuing ecosystem and economic services across land use scenarios in the Prairie Pothole Region of the Dakotas USA. Ecol. Econ..

[bib0145] Geerling-Eiff F.A., Van Der Meulen H.A.B. (2008). Bedrijfsbeëindiging in de land- en tuinbouw: Op een kruispunt en dan?.

[bib0150] Hermans C., Erickson J., Noordewier T., Sheldon A., Kline M. (2007). Collaborative environmental planning in river management: An application of multi-criteria decision analysis in the White River Watershed in Vermont. J. Environ. Manage..

[bib0155] Hilferink M., Rietveld P. (1999). Land use scanner: An integrated GIS based model for long-term projections of land use in urban and rural areas. J. Geogr. Syst..

[bib0160] Ianni E., Geneletti D. (2010). Applying the ecosystem approach to select priority areas for forest landscape restoration in the Yungas, Northwestern Argentina. Environ. Manage..

[bib0165] Janssen R. (2001). On the use of multi‐criteria analysis in environmental impact assessment in The Netherlands. JMCDA.

[bib0170] Jenkins W.A., Murray B.C., Kramer R.A., Faulkner S.P. (2010). Valuing ecosystem services from wetlands restoration in the Mississippi Alluvial Valley. Ecol. Econ..

[bib0175] Jongman R.H.G., Külvik M., Kristiansen I. (2004). European ecological networks and greenways. Landsc. Urban Plan..

[bib0180] Kamerbrief (8 March 2007). Kamerbrief inzake lange termijn discontovoet door de Minister van Financiën (Letter to the Dutch parliament by the Minister of Finance concerning the long term discount rate).

[bib0185] Keeney R.L., Raiffa H. (1976). Decisions with multiple objectives: preferences and value trade-offs.

[bib0190] Keeney R.L. (1996). Value-focused thinking: Identifying decision opportunities and creating alternatives. Eur. J. Oper. Res..

[bib0195] Langers F., Buijs A.E., de Vries S., Farjon J.M.J., Van Hinsberg A., Van Kampen P., Van Tol S., Sijtsma F.J. (2013). Potenties van de Hotspotmonitor om de graadmeter Landschap te verfijnen.

[bib0200] Leneman H., Schouten A.D., Verburg R.W. (2010). Varianten van natuurbeleid: voorbereidende kostenberekeningen.

[bib0205] Leneman H., Verburg R.W., Van Der Heide C.M., Schouten A.D. (2013). Kosten en Baten van terrestrische natuur - Methoden en resultaten.

[bib0210] Luttik J. (2000). The value of trees: water and open space as reflected by house prices in the Netherlands. Landsc. Urban Plan..

[bib0215] Mea, Sarukhán J., Whyte A. (2005). Ecosystems and human well-being biodiversity synthesis. Synthesis reports.

[bib0220] Mendoza G.A., Martins H. (2006). Multi-criteria decision analysis in natural resource management: A critical review of methods and new modelling paradigms. Forest Ecol. Manag..

[bib0225] Mnp (2006). Welvaart en Leefomgeving.

[bib0230] Mooney H.A. (2010). The ecosystem-service chain and the biological diversity crisis. Philos. Trans. R. Soc. B: Biol. Sci..

[bib0235] Mustajoki J., Saarikoski H., Marttunen M., Ahtikoski A., Hallikainen V., Helle T., Hyppönen M., Jokinen M., Naskali A., Tuulentie S., Varmola M., Vatanen E., Ylisirni A. (2011). Use of decision analysis interviews to support the sustainable use of the forests in Finnish Upper Lapland. J. Environ. Manage..

[bib0240] Nabuurs G.J., Schelhaas M.J. (2002). Carbon profiles of typical forest types across Europe assessed with CO2FIX. Ecol. Indic..

[bib0245] Nbtc-Nipo (2013). ContinuVrijeTijdsonderzoek (CVTO).

[bib0250] Nelson E., Mendoza G., Regetz J., Polasky S., Tallis H., Cameron D.R., Chan K.M.A., Daily G.C., Goldstein J., Kareiva P.M., Lonsdorf E., Naidoo R., Ricketts T.H., Shaw M.R. (2009). Modelling multiple ecosystem services, biodiversity conservation, commodity production, and trade-offs at landscape scales. Front. Ecol. Environ..

[bib0255] Nicholls S., Crompton J.L. (2005). The impact of greenways on property values: Evidence from Austin, Texas. J. Leisure Res..

[bib0260] Oikonomou V., Dimitrakopoulos P.G., Troumbis A.Y. (2011). Incorporating ecosystem function concept in environmental planning and decision making by means of multi-criteria evaluation: The case-study of Kalloni, Lesbos, Greece. J. Environ. Manage..

[bib0265] Pbl (2008). Natuurbalans 2008.

[bib0270] Pbl (2008). Kwaliteit voor later.

[bib0275] Pbl (2011). Nature Outlook 2010-2040. Nature and landscape in 2040: Development visions.

[bib0280] Pbl (2012). Balans van de Leefomgeving 2012.

[bib0285] Pbl (2013). Natuurverkenning 2010-2040. Achtergrondrapport [Nature Outlook 2010-2040-Background report].

[bib0290] Polasky S., Nelson E., Camm J., Csuti B., Fackler P., Lonsdorf E., Montgomery C., White D., Arthur J., Garber-Yonts B., Haight R., Kagan J., Starfield A., Tobalske C. (2008). Where to put things? Spatial land management to sustain biodiversity and economic returns. Biol. Conserv..

[bib0295] Poudyal N.C., Hodges D.G., Merrett C.D. (2009). A hedonic analysis of demand for and benefit of urban recreation parks. Land Use Policy.

[bib0300] Pouwels R. (2000). LARCH: een toolbox voor ruimtelijke analyses van een landschap.

[bib0305] Pouwels R., Van Eupen M., Kuipers H. (2011). Meta-Natuurplanner 2.0.

[bib0310] Pouwels R., Van Eupen M., Van Adrichem M.H.C., Knegt D.E.B., Van Der Greft J.G.M. (2016). MetaNatuurplanner v2.0.

[bib0315] Reichert P., Langhans S.D., Lienert J., Schuwirth N. (2015). The conceptual foundation of environmental decision support. J. Environ. Manage..

[bib0320] Reijnen R., Hinsberg Van A., Lammers W., Sanders M., Loonen W., de Jong T.M., de Dekker J.N.M., Posthoorn R. (2007). Optimising the Dutch National Ecological Network. Landscape ecology in the Dutch context: Nature, town and infrastructure.

[bib0325] Roodbol-Mekkes P.H., Van Der Valk A.J., Altes W.K.K. (2012). The Netherlands spatial planning doctrine in disarray in the 21st century. Env. Plan. A.

[bib0330] Schelhaas M.J., Van Wijk M.N., Nabuurs G.J. (2002). Koolstofvastlegging in bossen: Een kans voor de boseigenaar?. Nederlands bosbouwtijdschrift.

[bib0335] Seghezzo L., Vlante J.N., Paruelo J.M., Somma D.J., Buliubasich E.C., RODRíguez H.E., Gagnon S., Hufty M. (2011). Native forests and agriculture in Salta (Argentina): Conflicting visions of development. J. Env. Dev..

[bib0340] Sijtsma F.J. (2006). Project evaluation, sustainability and accountability: Combining cost-benefit analysis and multi-criteria analysis.

[bib0345] Sijtsma F.J., Heide C.M.J.V.D., Hinsberg A.V., Hull A., Alexander E., Khakee A., Woltjer J. (2011). Biodiversity and decision-support: Integrating CBA and MCA. Evaluation for participation and sustainability in planning.

[bib0350] Sijtsma F.J., Daams M.N., Farjon H., Buijs A.E. (2012). Deep feelings around a shallow coast. A spatial analysis of tourism jobs and the attractivity of nature in the Dutch Wadden area. Ocean and Coastal Manage..

[bib0355] Sijtsma F.J., de Vries S., Van Hinsberg A., Diederiks J. (2012). Does ‘grey’ urban living lead to more ‘green’ holiday nights? A Netherlands Case Study. Landsc. Urban Plan..

[bib0360] Sijtsma F.J., Farjon H., Van Tol S., Van Hinsberg A., Van Kampen P., Buijs A.E., Heijman W., Heide C.M.J.V.D. (2013). Evaluation of landscape changes: Enriching the economist’s toolbox with the Hotspotindex, Chapter 8. The Economic Value of Landscapes.

[bib0365] Sijtsma F.J., Heide C.M.J.V.D., Van Hinsberg A. (October 2013). Beyond monetary measurement: How to evaluate projects and policies using the ecosystem services framework. Environ. Sci. Policy.

[bib0370] Sollie S. (2007). Littoral zones in shallow lakes: Contribution to water quality in relation to water level regime.

[bib0375] Steinhäusser R., Siebert R., Steinführer A., Hellmich M. (2015). National and regional land-use conflicts in Germany from the perspective of stakeholders. Land Use Policy.

[bib0380] Strijker D., Sijtsma F.J., Wiersma D. (2000). Evaluation of nature conservation: An application to the Dutch Ecological Network. Environ. Resour. Econ..

[bib0385] Tang B.S., Wong S.W., Lee A.K.W. (2007). Green belt in a compact city: A zone for conservation or transition?. Landsc. Urban Plan..

[bib0390] Teeb, (2010). The economics of ecosystems and biodiversity mainstreaming the economics of nature: A synthesis of the approach, conclusions and recommendations of TEEB. TEEB.

[bib0395] Teeb, (2008). An interim report. TEEB.

[bib0400] Teel T.L., Manfredo M.J. (2010). Understanding the diversity of public interests in wildlife conservation. Conserv. Biol..

[bib0405] Tolkamp W., Van Den Berg C., Nabuurs G., Olsthoorn A. (2007). Kwantificering van beschikbare biomassa voor bio-energie uit Staatsbosbeheerterreinen.

[bib0410] Turnhout E., Hisschemöller M., Eijsackers H. (2007). Ecological indicators: Between the two fires of science and policy. Ecol. Indic..

[bib0415] Tzoulas K., Korpela K., Venn S., Yli-Pelkones V., Kazmierczak A., Niemela J., James P. (2007). Promoting ecosystem and human health in urban areas using Green Infrastructure: A literature review. Landsc. Urban Plan..

[bib0420] Van Der Bilt W.G.M., de Knegt B., Van Hinsberg A., Clement J. (2012). Van visie tot kaartbeeld; de kijkrichtingen ruimtelijk uitgewerkt: achtergronddocument bij Natuurverkenning 2011.

[bib0425] Van Der Windt H.J. (2012). Biologists bridging science and the conservation movement: The rise of nature conservation and nature management in the Netherlands, 1850-1950. Environ. Hist..

[bib0430] Van Hinsberg A., Van Der Bilt W.G.M., de Knegt B., Sijtsma F.J., Leneman H. (2011). Modelgebruik in de Natuurverkenning 2010-2040: De uitdagingen van het natuurbeleid geschetst en doorgerekend. Landschap.

[bib0435] Van Oostenbrugge R., Van Egmond P., Jorritsma I. (2010). Natuur als luxe of noodzaak: Natuurbeleid in beweging (in Dutch). De Levende Natuur.

[bib0440] Vessey I. (1991). Cognitive fit: A theory‐based analysis of the graphs versus tables literature. Decis. Sci..

[bib0445] Verboom J., Foppen R., Chardon P., Opdam P.F.M., Luttikhuizen P. (2001). Introducing the key patch approach for habitat networks with persistent populations: An example for marshland birds. Biol. Conserv..

[bib0450] Visser P., Van Dam F., Noorman N. (2006). De prijs van de plek: Woonomgeving en woningprijs. NAi Uitgevers.

[bib0455] Viglizzo E.F., Paruelo J.M., Laterra P., Jobbágy E.G. (2012). Ecosystem service evaluation to support land-use policy. Agric. Ecosyst. Environ..

[bib0460] Vonk M., Vos C.C., Van Der Hoek D.C.J. (2010). Adaptatiestrategie voor een klimaatbestendige natuur.

[bib0465] Wertenbroch K., Skiera B. (2002). Measuring consumers’ willingness to pay at the point of purchase. J. Mark. Res..

[bib0470] Willcox W.B. (1975). The Papers of Benjamin Franklin, vol. 19, January 1 through December 31, 1772.

[bib0475] Wolfslehner B., Seidl R. (2010). Harnessing ecosystem models and multi-criteria decision analysis for the support of forest management. J. Environ. Manage..

[bib0480] Zeleny M. (2011). Multiple criteria decision making (MCDM): From paradigm lost to paradigm regained?. JMCDA.

[bib0485] Zhang Y.J., Wei Y.M. (2010). An overview of current research on EU ETS: Evidence from its operating mechanism and economic effect. Appl. Energy.

